# The moderating role of central adiposity, sex and age, on the association between animal- and plant-based protein intake and the 20-year cumulative incidence of type 2 diabetes: the ATTICA cohort study (2002–2022)

**DOI:** 10.1007/s00394-026-03995-9

**Published:** 2026-06-06

**Authors:** Ioanna Kechagia, Fotios Barkas, Mary Yannakoulia, Evangelos Liberopoulos, Petros P. Sfikakis, Christos Pitsavos, Demosthenes Panagiotakos

**Affiliations:** 1 Department of Nutrition and Dietetics, School of Health Sciences & Education, Harokopio University, 17676 Athens, Greece; 2 Department of Clinical Dietetics-Nutrition, HYGEIA Hospital, 15123 Athens, Greece; 3 Department of Internal Medicine, Medical School, University of Ioannina, 45500 Ioannina, Greece; 4 First Department of Propaedeutic and Internal Medicine, Medical School, National and Kapodistrian University of Athens, Laiko General Hospital, 11527 Athens, Greece; 5 First Cardiology Clinic, Medical School, National and Kapodistrian University, Athens, Greece

**Keywords:** Protein intake, Type 2 diabetes, Animal-based protein, Plant-based protein, Metabolic status

## Abstract

**Purpose:**

The aim of this study was to evaluate the role of central adiposity, sex and age, on the association between total-animal and total-plant based protein and the 20-year cumulative incidence of type 2 diabetes (T2D), among apparently healthy adults participating in the ATTICA cohort study (2002–2022).

**Methods:**

The present analysis included data from 2,000 individuals free of atherosclerotic cardiovascular disease and T2D at baseline (age 43 ± 13 years; 51% women). Total, animal-based, and plant-based protein intake were measured, through a validated food frequency questionnaire.

**Results:**

The 20-year cumulative incidence of T2D was 26.3% (95%CI [24.4, 28.3%]). Total red meat intake was consistently associated with increased incidence of T2D in both sexes, with RRs ranging from 1.08 to 1.11 per 30 g/day increment (*p*’s < 0.05), after adjustment for confounders. Among females, a higher intake of animal-based protein was significantly associated with increased incidence of T2D (RR 1.24, 95%CI [1.04, 1.48]), with no significant associations among males. The adverse association between animal-based protein intake and T2D was strongest among females with increased WHR (RR 2.06, 95%CI [1.21, 3.49]); no significant association was observed in males, regardless of WHR status. Among younger females (< 65 years) with increased WHR, the association between animal-based protein and T2D incidence was equally strong (RR 2.06, 95%CI [1.21, 3.49]), and plant-based protein intake retained a highly significant protective association (RR 0.24, 95%CI [0.08, 0.68]).

**Conclusions:**

Long-term animal protein intake, especially red meat, increases the likelihood of developing T2D, particularly in women with central obesity; plant protein appears protective in metabolically vulnerable subgroups.

## Introduction

Type 2 diabetes (T2D) has emerged as one of the most pressing public health challenges of the 21st century, with its global burden projected to affect over 700 million adults by 2045 [1]. While the etiology of T2D is multifactorial, encompassing genetic, behavioral, and environmental factors, diet remains a central modifiable determinant in both the development and progression of the disease. Among dietary components, the role of protein intake—both in terms of quantity and source—has received increasing attention in recent years, with conflicting results [2].

Proteins from animal and plant sources differ significantly in their amino acid composition, metabolic effects, and accompanying nutrients, such as saturated fat, cholesterol, fiber, and phytochemicals. These differences may have divergent implications for glucose homeostasis, insulin sensitivity, and β-cell function [3]. Observational studies have consistently linked higher intake of red and processed meats with increased T2D incidence, potentially due to their saturated fat, heme iron, and advanced glycation end product content [4–5]. Conversely, plant-based protein sources—such as legumes, nuts, and whole grains—are often embedded within dietary patterns rich in fiber, antioxidants, and unsaturated fats, and the substitution of animal with plant protein, has been associated with improved insulin sensitivity and lower diabetes incidence [6–7].

Beyond dietary patterns, individuals’ characteristics may modify the association between protein intake sources and T2D incidence. Central adiposity, commonly assessed through the waist-to-hip ratio (WHR), has been strongly associated with insulin resistance and T2D incidence, independently of body mass index (BMI) [8]. It remains unclear whether the metabolic consequences of protein source differ according to adiposity distribution. Furthermore, sex-specific and age-related hormonal, physiological, and lifestyle differences may influence both dietary behaviors and diabetes pathophysiology, potentially moderating the effects of diet on T2D development [9–10].

Despite accumulating evidence, few prospective cohort studies have examined the long-term association between animal- and plant-based protein intake and T2D incidence, while concurrently considering the moderating roles of central adiposity, sex, and age. The ATTICA study, a population-based prospective cohort initiated in 2002 in the greater metropolitan area of Athens, Greece, provides a unique opportunity to address these gaps. With detailed dietary and clinical assessments, over a 20-year follow-up period, the study enables robust investigation into how habitual intake of protein from distinct sources relates to long-term diabetes incidence within diverse population strata. Therefore, the present study aimed to investigate the association between total, animal-based and plant-based protein intake and the 20-year cumulative incidence of T2D in the ATTICA cohort.

## Materials and methods

### Study design

The ATTICA study is an ongoing, prospective, population-based cohort study initiated in 2001–2002 in the metropolitan region of Attica, Greece. Its primary objective is to evaluate the longitudinal relationships between a wide range of sociodemographic, anthropometric, lifestyle, clinical, biochemical, and psychological parameters and the incidence of cardiometabolic disorders, including T2D and cardiovascular disease (CVD). Follow-up assessments were conducted at 5, 10, and 20 years (i.e., in 2006, 2012, and 2022, respectively). Comprehensive details on the study design, sampling methodology, and operational framework have been previously reported in detail [11–12].

### Sample and sampling procedure

From the 4,056 individuals who were invited to participate in the study, a total of 3,042 adults consented and were enrolled (participation rate: 75%). The cohort comprised 1,514 men (mean age: 43 ± 13 years; range: 18–87 years) and 1,528 women (mean age: 43 ± 13 years; range: 18–89 years). Exclusion criteria included a prior history of CVD, chronic viral infections, and institutionalization. For the current analysis, an additional 212 participants with a prior diagnosis of T2D or with baseline fasting glucose levels meeting diagnostic criteria for T2D were excluded. Participant recruitment employed a stratified random sampling strategy based on age and sex distributions within the Attica region. Standardized clinical assessments were performed by trained healthcare professionals—including cardiologists, nurses, general practitioners, and dietitians—either at the participants’ homes or workplaces.

### Follow-up and outcome assessment

At the 20-year follow-up in 2022, complete data on T2D status were available for 2,000 participants without baseline diabetes (follow-up rate: 71%). This subsample included 974 men and 1,026 women with similar age distributions (mean age: 43 ± 13 years at baseline). Incident T2D was ascertained according to the American Diabetes Association criteria—fasting plasma glucose > 125 mg/dL and/or use of antidiabetic medications [13]. Comparative analyses revealed no statistically significant differences in age or sex distribution between baseline and follow-up participant groups (*p* > 0.10).

### Ethical considerations

The study was conducted in accordance with the Declaration of Helsinki ethical principles. The study protocol received ethical approval from the Ethics Committee of the First Cardiology Department, National and Kapodistrian University of Athens (approval ID: #017/01.05.2001), and from the Ethics Committee of Harokopio University (approval ID: #38/29.03.2022).

### Data collection and measurements

Baseline assessments encompassed a range of sociodemographic, lifestyle, psychological, anthropometric, clinical, and biochemical variables, following rigorous standardized procedures.

Dietary intake was assessed via a validated semi-quantitative food frequency questionnaire (FFQ), which captured habitual intake in servings per day or per week. Specifically, participants provided information on their habitual dietary intake over the preceding 12 months through a FFQ [11]. Animal-based protein intake was assessed from a combination of dietary sources typically obtained from animals. This category included red meat and meat products, such as beef, lamb, and pork, including both fresh and possibly processed forms, poultry, including chicken, turkey, fish, encompassing both oily and lean varieties, dairy products, such as milk, yogurt, and cheese, and eggs, often consumed in a variety of forms including whole eggs and dishes where eggs are a major component. Plant-based protein intake was estimated from commonly consumed plant-derived foods that provide moderate to high amounts of protein. This category included legumes, such as lentils, beans, chickpeas, and peas, vegetables, i.e., leafy greens and root vegetables, and nuts. Both animal- and plant-based protein intake was transformed in g/day using food composition tables; total protein intake was determined by adding animal and plant-based protein intake.

Anthropometric indices, including BMI and WHR, included measurements of body weight (kg), height (m), waist and hip circumferences (cm), which were obtained following established protocols. BMI was computed as weight (kg) divided by height squared (m²), with overweight and obesity defined as BMI values of 25–29.9 kg/m² and ≥ 30 kg/m², respectively [14], and WHR was derived by dividing waist circumference by hip circumference. Central obesity, as defined by the World Health Organization (WHO), was assessed using the waist-to-hip ratio (WHR). According to WHO criteria, a WHR greater than 0.90 in men and greater than 0.85 in women indicates central (abdominal) obesity [15].

### Statistical analysis

Continuous variables were expressed as mean ± standard deviation (SD) (all variables were normally distributed). Comparisons of continuous variables between participants who developed T2D and those who did not were performed using independent-samples t-tests, where appropriate. The primary outcome of interest—the 20-year cumulative incidence of T2D—was calculated as the proportion of new T2D cases identified at the 20-year follow-up relative to the number of participants at baseline who were free of the disease. To investigate associations between animal- and plant- based protein intake and T2D incidence, multivariable logistic regression analyses were conducted. Odds ratios (ORs) with corresponding 95% confidence intervals (95% CIs) were calculated and considered as approximations of relative risks (RRs). Separate models were estimated for each source of protein intake, adjusting for potential confounding factors, including: age, sex, BMI, family history of T2D, and total protein intake. Survival analysis models (e.g., Cox proportional hazards) were not employed due to the lack of precise information regarding the timing of T2D onset in most cases. Therefore, logistic regression was deemed methodologically appropriate for assessing the cumulative incidence over the study period. All analyses were performed using STATA statistical software (version 17; MP & Associates, Sparta, Greece). Only complete cases were included in the final models; no imputation procedures were applied for missing data. Statistical significance was set at a two-tailed p-value < 0.05.

## Results

### Cumulative T2D incidence at 20-year follow-up

During the 2002–2022 period, 526 new cases of T2D were recorded out of the 2,000 participants, resulting in a cumulative incidence rate of 26.3% (21.4% for females and 31.4% for males). Details regarding factors associated with the development of T2D have been presented in a previous publication of the study [12].

### Total, animal-based, and plant-based protein intake and 20-year T2D incidence

In Table 1, weekly frequency of animal- and plant-based protein intake is presented, overall and stratified by 20-year T2D incidence. As observed, participants who developed T2D during the 20-year follow-up reported significantly higher red meat intake compared to those who did not develop T2D. Other animal protein sources (poultry, fish, dairy, and eggs) did not differ significantly between groups. Similarly, plant-based protein sources (legumes, vegetables, and nuts) and total protein intake showed no significant differences.

**Table 1:** Protein intake, in terms of animal-based, and of plant-based protein intake of the ATTICA study participants (*n* = 2,000), both in total and stratified according to the 20-year cumulative incidence of T2D; the ATTICA study (2002–2022)

Protein Intake	Total sample (*N* = 756)	T2D during 20-year FU		*p*-value
		Yes (*N* = 210)	No (*N* = 546)	
Sources of animal-based protein				
Total red meat intake, servings/week	4.7 (2.4)	5.0 (2.6)	4.6 (2.3)	0.016
Total poultry intake, servings/week	1.3 (0.9)	1.3 (0.8)	1.3 (0.9)	0.707
Total fish intake, servings/week	2.2 (1.3)	2.2 (1.3)	2.2 (1.3)	0.982
Total dairy intake, servings/week	11.8 (5.1)	11.8 (5.5)	11.8 (4.9)	0.964
Total egg intake, servings/week	1.1 (1.0)	1.1 (1.1)	1.1 (1.0)	0.826
Sources of plant-based protein				
Total legumes intake, servings/week	5.0 (2.8)	5.2 (2.5)	5.0 (2.8)	0.353
Total vegetables intake, servings/week	33.9 (13.9)	35.3 (14.7)	33.3 (13.5)	0.087
Total nuts intake, servings/week	1.6 (1.5)	1.6 (1.7)	1.5 (1.4)	0.584
Total protein intake, servings/week	15.6 (5.3)	16.6 (5.7)	15.4 (5.2)	0.071
Total animal-based protein intake, g/day	50 (14)	52 (16)	49 (13)	0.399
Total plant-based protein intake, g/day	34 (12)	38 (13)	32 (12)	0.075
*P*-value was based on the independent samples t-test (normally distributed continuous variables). Abbreviations: *T*2*D* Type 2 diabetes, *FU* Follow-up, *SD* Standard deviation.				

Multi-adjusted models were applied to evaluate the association between total protein intake, and the 20-year cumulative incidence of T2D, to account for potential residual confounding **(**Table 2**)**. Red meat intake was still significantly associated with increased T2D incidence. In *Model C* (i.e., adjusted for age, sex, BMI, family history of T2D, and total protein intake), each 30 g/day increment in red meat intake was associated with 11% higher odds of T2D. Both animal-based protein and plant-based protein intake showed non-significant associations with T2D incidence.

**Table 2:** Multi-adjusted logistic regression models evaluating the association between total protein intake, in terms of animal-based, and of plant-based protein intake of the ATTICA study participants (*n* = 2,000), and the 20-year cumulative incidence of T2D; the ATTICA study (2002–2022)

Models for: (per 30 g increment/day)	Model A: Nested model	Model B (naive): Model A, plus age and sex	Model C: Model B plus BMI, family history of T2D, and total protein intake
Total red meat intake	1.08 (1.01, 1.15)	1.09 (1.02, 1.16)	1.11 (1.02, 1.07)
Total poultry intake	1.04 (0.86, 1.25)	1.12 (0.93, 1.36)	1.10 (0.81, 1.50)
Total fish intake	0.99 (0.89, 1.12)	0.99 (0.88, 1.11)	1.18 (0.92, 1.51)
Total dairy intake	0.99 (0.97, 1.03)	1.02 (0.99, 1.05)	1.02 (0.97, 1.08)
Total egg intake	1.02 (0.87, 1.20)	1.01 (0.92, 1.27)	0.99 (0.78, 1.27)
Total legumes intake	1.03 (0.97, 1.09)	1.01 (0.95, 1.07)	0.96 (0.87, 1.06)
Total vegetables intake	1.01 (0.99, 1.02)	1.01 (0.99, 1.02)	0.99 (0.93, 1.06)
Total nuts intake	1.03 (0.93, 1.14)	1.02 (0.91, 1.13)	1.03 (0.88, 1.20)
Total animal-based protein intake	1.05 (0.94, 1.18)	1.14 (1.01, 1.28)	1.19 (0.99, 1.43)
Total plant-based protein intake	1.03 (0.99, 1.06)	1.02 (0.99, 1.05)	0.72 (0.45, 1.08)

RRs and their corresponding 95%CIs were obtained through logistic regression analysis as proxy of Odds Ratios. Fully adjusted model included: age (years), sex (men vs.women), body mass index (kg/m), family history of T2D (yes/no), and total protein intake (servings/week). Abbreviations: *RR* Relative risk, *CI* Confidence interval, *BMI* Body mass index, *T*2*D T*ype 2 diabetes.

### Evaluating the role of sex in the association between animal-based, and plant-based protein intake and the 20-year cumulative T2D incidence

A significant interaction between protein intake (animal- and plant-based) and sex was observed for 20-year T2D incidence (*p* for interaction < 0.001). Stratified analysis showed that in females, higher total animal-based protein intake was significantly associated with a 24% higher odds of developing T2D (Model B), and this association remained significant (29% higher odds of developing T2D) in the fully adjusted model. Moreover, total plant-based protein intake among females was inversely associated with T2D incidence (Model C). In males, neither total animal-based nor total plant-based protein intake showed significant associations with T2D in any model **(**Table 3**).**

**Table 3:** Multi-adjusted logistic regression models evaluating the association between total protein intake, in terms of animal-based, and of plant-based protein intake of the ATTICA study participants (*n* = 2,000), stratified by sex, and the 20-year cumulative incidence of T2D; the ATTICA study (2002–2022)

Models for: (per 30 g increment/day)	Model A: Nested model	Model B (naive): Model A, plus age	Model C: Model B plus BMI, family history of T2D, and total protein intake
Females			
Total animal-based protein intake	1.17 (0.99, 1.38)	1.24 (1.04, 1.48)	1.29 (0.95, 1.76)
Total plant-based protein intake	1.05 (1.01, 1.11)	1.04 (0.99, 1.10)	0.60 (0.32, 1.11)
Males			
Total animal-based protein intake	0.97 (0.82, 1.13)	1.06 (0.89, 1.25)	1.11 (0.84, 1.47)
Total plant-based protein intake	1.01 (0.97, 1.05)	1.01 (0.96, 1.05)	0.81 (0.47, 1.41)

RRs and their corresponding 95%CIs were obtained through logistic regression analysis as proxy of Odds Ratios. Fully adjusted model included: age (years), , body mass index (kg/m^2^), family history of T2D (yes/no), and total protein intake (servings/week). Abbreviations: *RR* Relative risk, *CI* Confidence interval, *BMI* Body mass index, *T*2*D* Type 2 diabetes.

### Evaluating the role of central adiposity in the association of protein intake and 20-year cumulative T2D incidence

A highly significant interaction between protein intake (- )animal- and plant-based) and WHR was also observed for 20-year T2D incidence (*p* for interaction < 0.001). Stratified analysis revealed that the association of animal-based protein intake with T2D was strongest (a 106% higher odds of developing T2D) among females with increased WHR, as compared to those with WHR within normal range (Table 4, Model C). Conversely, total plant-based protein intake was significantly protective (a 76% lower odds of developing T2D) in the same group of participants (Table 4, Model C), as compared to females with normal WHR. Moreover, no significant associations between protein intake and T2D incidence were observed in all male subgroups according to WHR **(**Table 4**).**

**Table 4:** Multi-adjusted logistic regression models evaluating the association between total protein intake, in terms of animal-based, and of plant-based protein intake of the ATTICA study participants (*n* = 2,000), stratified by sex and waist to hip ratio, and the 20-year cumulative incidence of T2D; the ATTICA study (2002–2022)

Models for: (per 30 g increment/day)	Model A: Nested model	Model B (naive): Model A, plus age	Model C: Model B plus BMI, family history of T2D, and total protein intake
Females with increased waist to hip ratio			
Total animal-based protein intake	1.47 (1.08, 2.00)	1.50 (1.10, 2.05)	2.06 (1.21, 3.49)
Total plant-based protein intake	1.05 (0.97, 1.15)	1.05 (0.96, 1.14)	0.24 (0.08, 0.68)
Females with normal waist to hip ratio			
Total animal-based protein intake	1.06 (0.84, 1.33)	1.19 (0.92, 1.52)	0.99 (0.65, 1.52)
Total plant-based protein intake	1.06 (0.99, 1.13)	1.05 (0.98, 1.14)	1.01 (0.43, 2.37)
Males with increased waist to hip ratio			
Total animal-based protein intake	1.05 (0.78, 1.41)	1.10 (0.81, 1.50)	1.11 (0.69, 1.77)
Total plant-based protein intake	1.01 (0.94, 1.08)	0.99 (0.93, 1.07)	0.82 (0.32, 2.09)
Males with normal waist to hip ratio			
Total animal-based protein intake	0.95 (0.78, 1.15)	1.01 (0.82, 1.24)	1.17 (0.81, 1.68)
Total plant-based protein intake	1.01 (0.96, 1.06)	1.01 (0.96, 1.06)	0.73 (0.36, 1.51)

RRs and their corresponding 95%CIs were obtained through logistic regression analysis as proxy of Odds Ratios. Fully adjusted model included: age (years), , body mass index (kg/m^2^), family history of T2D (yes/no), and total protein intake (servings/week). Abbreviations: *RR* Relative risk, *CI* Confidence interval, *BMI* Body mass index, *T*2*D* Type 2 diabetes.

### Evaluating the role of age in the association of protein intake and 20-year cumulative T2D incidence

A significant interaction was observed between age, and protein intake groups on T2D incidence (*p* for interaction < 0.001). It was observed that among females younger than 65 years old with increased WHR, total animal-based protein intake was associated with 106% higher odds of T2D, while total plant-based protein intake was associated with 76% lower odds of T2D, even various adjustments made (Table 5). In all other subgroups studied (i.e., females with normal WHR or males, with or without normal WHR), no significant associations were found between protein intake and T2D incidence **(**Table 5**)**.

**Table 5:** Multi-adjusted logistic regression models evaluating the association between total protein intake, in terms of animal-based, and of plant-based protein intake of the ATTICA study participants aged < 65 years old (*n* = 1,883), stratified by sex, waist to hip ratio and the 20-year cumulative incidence of T2D; the ATTICA study (2002–2022)

Models for: (per 30 g increment/day)	Model A: Nested model	Model B (naive): Model A, plus age	Model C: Model B plus BMI, family history of T2D, and total protein intake
Females with increased waist to hip ratio			
Total animal-based protein intake	1.47 (1.08, 2.00)	1.50 (1.10, 2.05)	2.06 (1.21, 3.49)
Total plant-based protein intake	1.05 (0.97, 1.15)	1.05 (0.96, 1.14)	0.24 (0.08, 0.68)
Females with normal waist to hip ratio			
Total animal-based protein intake	1.06 (0.84, 1.33)	1.19 (0.92, 1.52)	0.99 (0.65, 1.52)
Total plant-based protein intake	1.06 (0.99, 1.13)	1.05 (0.98, 1.14)	1.01 (0.43, 2.37)
Males with increased waist to hip ratio			
Total animal-based protein intake	1.05 (0.78, 1.41)	1.10 (0.81, 1.50)	1.11 (0.69, 1.77)
Total plant-based protein intake	1.01 (0.94, 1.08)	0.99 (0.93, 1.07)	0.82 (0.32, 2.09)
Males with normal waist to hip ratio			
Total animal-based protein intake	0.95 (0.78, 1.15)	1.01 (0.82, 1.24)	1.17 (0.81, 1.68)
Total plant-based protein intake	1.01 (0.96, 1.06)	1.01 (0.96, 1.06)	0.73 (0.36, 1.51)

RRs and their corresponding 95%CIs were obtained through logistic regression analysis as proxy of Odds Ratios. Fully adjusted model included: age (years), , body mass index (kg/m^2^), family history of T2D (yes/no), and total protein intake (servings/week). Abbreviations: *RR* Relative risk, *CI* Confidence interval, *BMI* Body mass index, *T*2*D* Type 2 diabetes.

## Discussion

This 20-year prospective analysis from the ATTICA cohort study contributes novel insights into the complex relationship between dietary protein sources and the long-term incidence of T2D, highlighting the moderating roles of sex, central adiposity, and age. While prior research has reported the impact of total and source-specific protein intake on metabolic health, this is, to our knowledge, the first Mediterranean population-based study to comprehensively examine the joint effect of animal and plant protein intake on T2D incidence over two decades, stratified by key biological moderators such as WHR, sex, and age.

Consistent with previous evidence, higher intake of animal-based protein—particularly from red meat—was associated with significantly increased odds of developing T2D, independent of established confounders including age, sex, BMI, and family history of the disease. Higher intake of meat—particularly processed and unprocessed red meat—is associated with higher T2D incidence, according to the findings of a large-scale individual-participant meta-analysis from the InterConnect project, involving over 1.9 million adults across 31 global cohorts [16]. Specifically, a 50 g/day increment in red meat intake was associated with an 11% higher incidence of T2D, after adjusting for adiposity and other potential confounds, according to findings from a large prospective study [17]. These consistent results reinforce current dietary recommendations aimed at reducing red and processed meat intake to mitigate diabetes incidence. However, accordind to findings from a recent umbrella review, higher total protein intake may be associated with increased odds of developing T2D, although evidence for animal protein remains limited, and plant protein appears to have no significant effect [18].

Importantly, this study extends the literature by revealing that the adverse effects of animal protein intake were significantly more pronounced among women, particularly those with central obesity. Among females with elevated WHR, the odds of developing T2D were more than doubled with higher intake of animal-based protein. This finding remained robust even after further stratification by age, suggesting that visceral adiposity may amplify the deleterious metabolic effects of animal-derived nutrients, particularly among younger women. These results support prior evidence that the interplay between sex hormones, fat distribution, and systemic inflammation may modify the effect of dietary exposures on the development of T2D.

Several large-scale prospective cohort studies and meta-analyses have investigated the association between dietary protein intake and T2D incidence, with emerging evidence suggesting potential sex-specific differences in this relationship. Data from the Melbourne Collaborative Cohort Study indicated that higher intake of plant protein was inversely associated with the development of T2D in women, but not in men, suggesting possible sex-related biological interactions [19]. In contast, a recent review discussed observational studies linking high total and animal protein intake to an increased incidence of T2D, particularly in obese women, suggesting the role of branched-chain amino acids (BCAAs) in insulin resistance and T2D pathogenesis [20]. Similarly, analyses from the EPIC-InterAct study demonstrated a modestly elevated incidence of T2D with higher total and animal protein intake, with stronger associations observed among women, particularly those with obesity [21]. In line with the previous findings, our study demonstrated a significant positive association between the odds of developing T2D and the total animal-based protein intake, during a long-time frame, i.e., 20 years, among females with central adiposity and < 65 years old, while the above association remained non significant, concerning the subgroup of females with no central obesity, and among males independently of central adiposity. Moreover, the protective effect of plant-based protein intake on the 20-year T2D incidence remained significant, among females with central adiposity, comparable to other subgroups, after adjusting for various confounders.

Recent studies have elucidated the multifaceted role of estrogens in modulating insulin sensitivity, adipose tissue distribution, and inflammatory status, thereby influencing the development of T2D. Estrogens enhance insulin action by activating the PI3K-Akt-FoxO1 signaling pathway, leading to suppressed hepatic gluconeogenesis and improved glucose homeostasis. Moreover, estrogens promote a favorable adipose tissue distribution by encouraging subcutaneous fat deposition over visceral fat accumulation, which is associated with a lower likelihood of metabolic disorders. Additionally, estrogens exhibit anti-inflammatory properties by decreasing pro-inflammatory cytokine levels in adipose tissue, further contributing to enhanced insulin sensitivity. These mechanisms collectively underscore the protective effects of estrogens against the development of insulin resistance and T2D [22–24]. However, it is noteworthy that none of the aforementioned studies directly assessed circulating sex hormone levels or explored mechanistic interactions between sex hormones and dietary protein intake. This highlights a significant gap in the current literature and underscores the need for future research to examine hormonal modulation as a potential pathway through which dietary protein affects T2D development in a sex-specific manner.

Substituting animal protein with plant protein is associated with lower T2D incidence. Studies showed that replacing animal with plant-based sources such as legumes, nuts, or soy can reduce the likelihood of T2D by 15–35% [3, 25]. According to findings from a systematic review and meta-analysis, adherence to healthy plant-based dietary patterns—characterized by high consumption of vegetables, fruits, whole grains, and legumes, is associated with a lower likelihood of noncommunicable diseases, including T2D, CVD, cancer, and mortality. Conversely, unhealthy plant-based diets, defined by a high intake of refined grains, sugar-sweetened beverages, sweets, and highly processed plant-based foods, often characterized by low fiber and micronutrient content and high levels of added sugars and sodium, show positive associations with these adverse health outcomes [26]. Recent meta-analyses and cohort studies have shown that higher intake of plant protein is inversely associated with T2D incidence, with the strongest protective effects observed in metabolically vulnerable populations, such as those with obesity, prediabetes, insulin resistance, or inflammation [3, 27]. These findings suggest that plant-based dietary patterns rich in plant protein may confer greater metabolic benefits for vulnerable individuals by improving glycemic control and reducing insulin resistance. Such stratified protective effects have been previously underreported in large epidemiologic studies and point to a potential need for tailored dietary interventions. In our study, plant-based protein intake also showed a protective association, reaching significance among women with central obesity.

The biological plausibility of these findings is supported by several mechanisms. Animal protein, especially red and processed meat, often coexists with higher intake of saturated fat, heme iron, nitrates, and advanced glycation end products, all of which have been linked to insulin resistance and pancreatic β-cell dysfunction [28–29]. In contrast, plant protein sources are typically accompanied by dietary fiber, polyphenols, unsaturated fatty acids, and micronutrients such as magnesium, which have anti-inflammatory and insulin-sensitizing effects [30].

Furthermore, while earlier meta-analyses and pooled analyses have established the adverse effects of animal protein and the beneficial effects of plant protein [6, 31], our study uniquely demonstrates that these associations are not homogeneous across population strata. Instead, they are significantly moderated by sex, fat distribution, and age, supporting a more personalized approach to dietary recommendations for T2D prevention.

### Strengths and limitations

The present analysis is based on data from the ATTICA study, a large-scale, population-based, prospective cohort with a 20-year follow-up period, rendering it one of the few longitudinal investigations of T2D with extended temporal depth conducted globally. This prolonged follow-up enhances the study’s ability to capture the cumulative effects of dietary exposures on T2D incidence, over an extended period. Nonetheless, several methodological considerations should be acknowledged. First, as an observational study, the potential for residual confounding cannot be fully eliminated, despite comprehensive adjustment for a range of sociodemographic, lifestyle, and clinical covariates. Such unmeasured or imprecisely measured factors may partially account for the associations observed. Second, the absence of precise dates of T2D onset precluded the use of time-to-event analytical approaches and limited the ability to compute incidence rates. Third, dietary intake was assessed at baseline only; thus, any changes in dietary patterns, including protein source and quantity, over the follow-up period were not captured. This limitation may attenuate the observed associations and precludes examination of longitudinal dietary trajectories in relation to T2D development. Furthermore, approximately one-quarter of the initial cohort was lost to follow-up, raising concerns about potential attrition bias, although comparisons between retained and non-retained participants showed no substantial differences in baseline characteristics. However, the stratified analyses led to a substantial reduction in subgroup samples sizes, reducing the statistical power of the findings. Moreover, the moderating role of clinical and pre-clinical obesity was not investigated, according to the most recent definition of the Lancet Diabetes & Endocrinology Commission-so as to better capture metabolic vuklnerability [32].

Despite these limitations, the ATTICA study provides valuable epidemiological evidence on the interplay between dietary protein sources, central adiposity, and T2D development. Its extensive follow-up, representative Mediterranean population, and incorporation of both total and source-specific protein intake measures contribute meaningfully to the growing body of literature on precision nutrition and metabolic disease prevention. Another key strength of this study is its methodological approach: the long follow-up period of 20 years, combined with comprehensive dietary and anthropometric assessments and adjustment for a broad set of confounding variables, allows for robust inferences. Unlike most prior studies that primarily relied on BMI as an adiposity marker, this analysis used WHR to capture central obesity, a more potent predictor of cardiometabolic outcomes, thereby enhancing the granularity of metabolic profiling. Another key finding of this study is the identification of a significant interaction between dietary protein source and central adiposity—specifically that women with elevated WHR are disproportionately affected by high animal protein intake and particularly benefit from plant-based protein. This highlights a sex-specific and fat-distribution-dependent susceptibility to protein type in the pathogenesis of T2D, which has been insufficiently investigated in previous cohort studies.

## Conclusion

Long-term animal-based protein intake, particularly red meat, is associated with a higher likelihood of type 2 diabetes, especially among women with central obesity. In contrast, plant-based protein intake may confer protective benefits, particularly in metabolically vulnerable groups. These findings underscore the need to consider individual characteristics such as sex, age, and body fat distribution when developing dietary guidelines for diabetes prevention. Future research should explore underlying biological mechanisms and examine whether targeted dietary interventions can mitigate the development of diabetes in metabolically vulnerable subpopulations.

## Data Availability

The data presented in this study are available on request from the corresponding author. The data are not publicly available due to privacy restrictions.
